# 

**Published:** 1909-06-12

**Authors:** 


### ANSWERS TO CORRESPONDENTS.

Dr. D. M. Macdonald.—In reply to your request for the date of a paper on the use of Liquor Colchici for Gout, etc., we have consulted the indices of the past two volumes, and the file of recent issues in the current volume; but as we go to press we have so far failed to find any reference to the drug in question. The issue of February 15, 1908, contains Part II. of an article on "Acute Gout," which mentions the Acetic Extract of Colchicum but not the Liquor. We will inform you in the event of our discovering the desired reference.

D. E. A. (Highgate).—Your questions deal with matters of considerable general interest; but, since adequate replies to the specific point upon which you desire detailed information would occupy more space than is available in this column, we are answering your letter through the post. This, however, is a course which we cannot undertake to follow, even when (as in your case) stamps are enclosed, except under exceptional conditions.

Correspondents seeking replies to their queries would do well to add initials or a pseudonym to their signatures; since, even when a personal answer by post is especially solicited, it may be impossible or inadvisable for us to comply by means of a private letter. Readers who fail to receive an answer to their queries through the post should invariably turn to this column in the ensuing issues of The Hospital, in case a public or semi-public reply, following their initials, should be printed amongst answers to correspondents. These answers usually appear immediately after current letters from readers under the inclusive heading "Editor's Letter Box," except in the case of matters appropriate only to the resident medical officers, when a reply from the Editor of the section may be published under that heading.—[Editor The Hospital.]

Messrs. J. and A. Churchill announce a new edition of "Minor Surgery and Bandaging," revised by Mr. Bilton Pollard, Surgeon to University College Hospital. The first edition of this work, by Mr. Christopher Heath, was issued forty-eight years ago. The present (fourteenth) edition has been enlarged by nearly a hundred pages, and contains many new figures and a frontispiece of a surgeon in aseptic operating costume. "An Atlas of Dental Extractions, with Notes on the Causes and Relief of Dental Pain," by Mr. C. E. Wallis, Assistant Dental Surgeon, King's College Hospital, is also announced by these publishers. The text will be supplemented with a series of illustrative plates.

### THE GRADUATES' MEDICAL SCHOOLS.

DIARY FOR THE WEEK, JUNE 14 to JUNE 19.

**ROYAL SOCIETY OF MEDICINE, 20 Hanover Square, W.**

**At 5 p.m.**

**June 17, Dermatological Section.**

Dr. Wilfrid Fox and Dr. Rolleston: "A case of leukæmic infiltration of the skin." And other cases.

**June 18, Section for the Study of Disease in Children.**

PROVINCIAL MEETING AT EDINBURGH.

**At 2.30 p.m.**

Demonstration of cases and specimens at the Edinburgh Royal Hospital for Sick Children.

**At 5 p.m.**

Meeting in the Lecture Theatre of the Children's Hospital. Papers will be read.

**MEDICAL GRADUATES' COLLEGE AND POLYCLINIC, 22 Chenies Street, W.C.**

**At 5.15 p.m.**

**June 14,** Mr. Lockhart Mummery, **The Diagnosis of Diseases of the Rectum and Anus.**

**June 15,** Dr. W. H. H. Tate, **Climacteric Hæmorrhage.**

**June 16,** Mr. Clinton Dent, **Recent Experiences with the Schaifer Method of Artificial Respiration.**

**June 17,** Mr. Edred Corner, **Affections of the Umbilicus.**

**THE POST-GRADUATE COLLEGE, West London Hospital, Hammersmith, W.**

**At 10 a.m.**

**June 14 and 17,** Surgical Registrar, **Demonstration.**

**June 18,** Medical Registrar, **Demonstration.**

**At 12 noon.**

**June 14,** Pathologist, **Pathological Demonstration.**

**At 12.15 p.m.**

**June 15** Dr. Pritchard, **Practical Medicine.**

**June 16 and 19,** Dr. Grainger Stewart, **Practical Medicine.**

**At 5 p.m.**

**June 14,** Mr. Bidwell, **Practical Surgery.**

**June 15,** Dr. Seymour Taylor, **Aortic Obstruction.**

**June 16,** Dr. Beddard, **Medicine—IV.**

**June 17,** Mr. Edwards, **Clinical Lecture.**

**June 18,** Mr. Harman, **"Pink Eye."**

**THE THROAT HOSPITAL, Golden Square, W.**

**At 5.30 p.m.**

**June 14,** Dr. Bond, **Acute and Chronic Inflammation of the Mastoid.**

**June 17,** Mr. Rose, **Intra-Cranial Complications: Symptoms and Diagnosis.**

**NORTH-EAST LONDON POST-GRADUATE COLLEGE, Prince of Wales's General Hospital, Tottenham, N.**

**At 4.30 p.m.**

**June 15,** Dr. G. G. Macdonald, **The Pathology of Cardiac Inflammations.**

**June 17,** Dr. F. H. Wallace, **Methods of Producing Local Anæsthesia.**

**LONDON SCHOOL OF CLINICAL MEDICINE, Seamen's Hospital, Greenwich, S.E.**

**At 3.15 p.m.**

**June 15,** Mr. Carless, **Fractures about the Ankle.**

**At 3.30 p.m.**

**June 16,** Mr. Cargill, **Glaucoma: Diagnosis and Treatment.**

**At 2.15 p.m.**

**June 18,** Dr. R. Bradford, **Some Varieties of Anæmia.**

**NATIONAL HOSPITAL FOR THE PARALYSED AND EPILEPTIC, Queen Sq., Bloomsbury, W.C.**

**At 3.30 p.m.**

**June 15,** Sir W. Gowers, **Tremor.**

**June 18,** Sir Victor Horsley, **Surgery of the Nervous System.**

THE HOSPITAL JUNE 12, 1909.

*Name* ..................................................

*Address* ..................................................

..................................................

This Coupon must accompany manuscript or contributions intended for The Hospital.

